# Efficacy of Adjunctive Antiseptic Lavage Solution in Managing Acute Hip/Knee Prosthetic Joint Infection: A Comparative Study in a Tertiary Revision Center

**DOI:** 10.1016/j.artd.2024.101593

**Published:** 2025-01-21

**Authors:** Jonathan Quinn, Bernard H. van Duren, Reshid Berber, Mark Higgins, Hosam E. Matar, Andrew R. Manktelow, Benjamin V. Bloch

**Affiliations:** a Nottingham Elective Orthopaedic Services, Nottingham University Hospitals NHS Trust, Nottingham, UK; b Leeds Institute of Rheumatic and Musculoskeletal Medicine, University of Leeds, Leeds, UK; c School of Medicine, University of Nottingham, Nottingham, UK

**Keywords:** Hip, Knee, PJI, DAIR, Infection, Bactisure

## Abstract

**Background:**

Debridement, antibiotics and implant retention (DAIR) procedure is well-established as a management option for acute periprosthetic joint infection (PJI). We investigated the infection eradication rates of DAIR procedures at our center using Bactisure wound lavage.

**Methods:**

A retrospective consecutive review of DAIR procedures for hip and knee PJI was conducted between 2018 and 2023 with a minimum 12-month follow-up at our tertiary revision arthroplasty center. Suitability for DAIR was determined at the multi-disciplinary team discussion. Revision procedures and patients with previous PJI were excluded. Patient, surgical, microbiological, and postoperative data (minimum 12 months) was reviewed. The use of Bactisure was in addition to routine surgical management.

**Results:**

During the study period, 76 DAIR procedures were performed (55 knees and 21 hips). Bactisure was used in 26 cases (20 knees and 6 hips). Overall, 6 of 26 Bactisure DAIRs failed (23%), while 14 of 50 non-Bactisure DAIRs failed (28%), which did not demonstrate statistical significance (*P* = .644). Subgroup analysis demonstrated no difference in knee DAIRs (*P* = .761) but a trend toward significance in hip DAIRs (*P* = .262). No adverse effects of Bactisure use were noted intraoperatively or postoperatively. DAIR failed in 50% of diabetic patients compared to 20% of nondiabetic patients (*P* = .015). Age, body mass index, and organism identification did not influence outcome.

**Conclusions:**

The addition of Bactisure to DAIR procedures did not demonstrate statistically significant improvement of successful eradication of infection, but a potential trend toward significance was noted in hip DAIRs. Diabetic patients failed DAIR in 50% of cases. The in-vivo outcomes of Bactisure use during DAIR procedures remain inconclusive.

## Introduction

Periprosthetic joint infection (PJI) is a recognized complication, being the second most common cause for revision in total hip arthroplasty and the most common indication for revision in total knee arthroplasty [[1], [2], [3], [4]]. The consequences of PJI are devastating with regard to patients’ quality of life, often necessitating repeated hospitalizations and invasive treatments, and are associated with increased health care costs and mortality [1,5,6]. Single-stage and 2-stage revision are well-established treatments for PJI [7,8]. However, these are significantly invasive procedures requiring the removal of implants and the use of complex revision components [9]. In specific circumstances, it is feasible to undertake a less invasive approach with debridement, antibiotics and implant retention (DAIR). DAIR retains the primary implant, exchanging out modular components where possible, aiming to preserve function and reduce morbidity [10]. Although well-established as a management option for acute PJI, reported success rates are varying widely from 39% to 85% [[10], [11], [12]].

Crucial to the success of DAIR is the eradication of the bacterial biofilm on the implant surfaces. Biofilm is an extracellularly produced polymeric matrix that shields bacteria from the host’s immune response and acts as a physical barrier to limit the penetration of antimicrobial agents [13]. Acetic acid (AA) is a weak organic acid that is active against gram-positive and gram-negative organisms [[14], [15], [16]]. Early studies showed that AA has an acceptable safety profile in vivo and was effective in eliminating a significant proportion of biofilms in vitro [14,17]. However, given that the use of AA or variations thereof is not in widespread use within the arthroplasty community, there is a paucity of information on its clinical effectiveness when used as part of a DAIR procedure. Bactisure wound lavage (Zimmer Biomet, Warsaw, IN, USA) is a commercially available antiseptic solution that combines the use of AA with ethanol, sodium acetate, benzalkonium chloride, and water and is increasingly being utilized as an adjunct in DAIR procedures. In vitro studies have demonstrated efficacy of Bactisure in biofilm reduction [[18], [19], [20]]. Andriollo et al. have reported on their series of 39 patients using Bactisure as an adjunct to DAIR procedures in hip and knee acute PJI with promising results [5]. However, no comparative studies have yet been published comparing the results of DAIR with Bactisure in comparison to “standard” DAIR.

The aim of this study was therefore to compare the outcomes of DAIR procedures at a high-volume revision arthroplasty center, with the use of Bactisure wound lavage to the previous standard of copious lavage and chlorhexidine wash. We hypothesized that Bactisure use would result in no improved infection eradication rate following DAIR procedures, compared to DAIR procedures conducted without Bactisure use.

## Material and methods

This was a retrospective study of all consecutive patients who underwent a DAIR procedure for hip and knee arthroplasty PJI between 2018 and 2023 inclusive. Institutional approval was granted prior to the commencement of the study. We identified our cohort through local prospective electronic databases and linkable data obtained from the National Joint Registry. Operative records, multi-disciplinary team records, and clinical notes are used to identify details of care.

### Inclusion criteria were

• Patients receiving care by subspecialist revision arthroplasty surgeons at our facility (a regional revision center).
• All patients had been deemed suitable for DAIR following revision multi-disciplinary team discussion including orthopaedic surgeons and microbiologists [21].
• A minimum follow-up of 12 months.

### Exclusion criteria were

• Patients with previous PJI (chronic infection).
• Patients who underwent removal of nonmodular implants (single or 1st stage revision procedures).

The use of Bactisure was in addition to routine surgical practice for DAIR procedures, which includes meticulous and thorough soft tissue debridement, removal of modular implants, copious lavage with 0.9% NaCl, and 2% chlorhexidine wash. Bactisure was used in our center from March 2021 onward and was used in the majority of DAIRs performed from this date, based on surgeon preference. The surgeons using Bactisure used it to wash the remaining implants and tissues following standard debridement and lavage then subsequently soaked the joint for 20 minutes with the tourniquet deflated. Following the Bactisure soak, the sterile field was reprepared/redraped before the joint and implants were lavaged with 0.9% NaCl and washed with 2% chlorhexidine prior to placing the new modular components and closure.

The primary outcome was failure of DAIR procedure, defined as recurrence of infection on microbiology or return to theater for further surgical intervention. Patients who received the addition of Bactisure were compared to those who did not have Bactisure used during DAIR (control group) for statistical analysis. Secondary outcomes included detection of adverse effects of Bactisure use, the impact of comorbidities on DAIR success, and the impact of duration since surgery on DAIR success.

Continuous descriptive statistics used means, median values, ranges, 95% confidence intervals, and graphical representations where appropriate. Contingency tables and either a chi-squared or Fisher’s exact test (when the number of events was small) were used to determine significance depending on sample size. The level of significance was taken to be *P* < .05. RStudio (version 2022.02.2) was used to perform the analyses.

## Results

**Figure 1:**
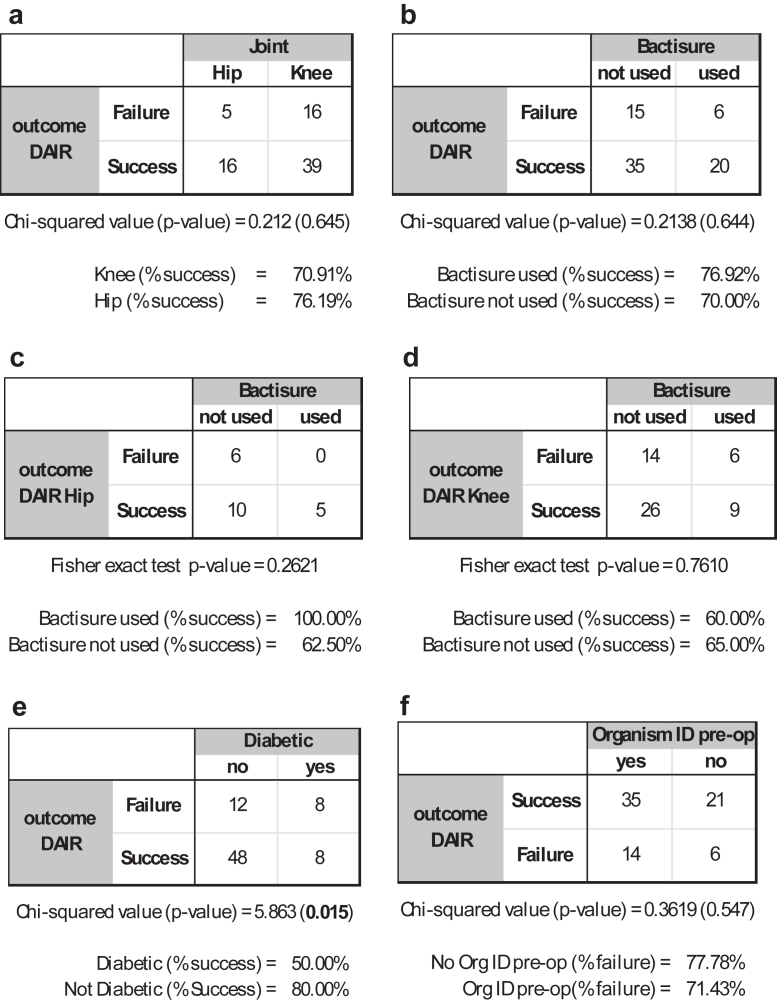
Contingency tables and either a chi-squared or Fisher’s exact test (when the number of events was small) *P*-values for (a) comparison between hip and knee; (b) comparison between standard and Bactisure groups; (c) comparison between standard and Bactisure groups in hips only; (d) comparison between standard and Bactisure groups in knees only; (e) comparison of diabetic vs nondiabetic; and (e) whether an organism was identified preoperatively or not.

During the study period, 76 DAIR procedures meeting the inclusion criteria were performed (55 knees and 21 hips). Bactisure was used in 26 cases (20 knees and 6 hips); see Table 1. No adverse effects attributable to Bactisure use were noted intraoperatively or postoperatively. Overall, 6 of 26 Bactisure DAIRs failed (23%), while 15 of 50 standard DAIRs failed (30%), which did not demonstrate statistical significance (*P* = .645); see Figure 1.

**Table 1:** Overview of collected demographic data and outcomes.

Parameter	Units	All	Standard	Bactisure
n (knees/hips)	-	76 (55/21)	50 (35/15)	26 (20/6)
Sex (m/f)	-	41/35	26/24	15/11
Mean age @ DAIR (range)	y	69.64 (24-96)	69.84 (24-95)	69.30 (40-96)
Mean BMI (range)	kg/m2	33.17 (7.72)	33.4 (19-59)	32.7 (19-50)
Mean days from primary (range)	d	1120 ('12-8210)	1216 (12-8210)	935 (13-4037)
ASA grade [1,2]	-	1-33-37-5	1-19-28-2	0-14-9-3
Diabetic (yes/no)	-	16/60 (21%)	12 (24%)	3 (12%)
Mean follow-up (range)	mo	38 (12-74)	46 (14-74)	22 (12-38)
Failed DAIR (knees and hips)	-	20 (26%)	15 (30%)	6 (23%)
Failed DAIR (knees)	-	16 (29%)	10 (29%)	6 (30%)
Failed DAIR (hips)	-	5 (24%)	5 (33%)	0 (0%)

ASA, American Society of Anesthesiologists; BMI, body mass index; DAIR, debridement, antibiotics, and implant retention.

Subgroup analysis of knee DAIRs demonstrated no statistically significant difference in outcome, where 6 of 20 DAIRs using Bactisure failed (30% failure), while 10 of 35 DAIRs without Bactisure failed (29% failure), *P* = .761. In hip DAIRs, Bactisure was used in 6 of 21 cases. Five out of 15 standard DAIRs failed (33.3% failure), while 0 out of 6 DAIRs using Bactisure failed (0% failure). While this data were not statistically significant (*P* = .262).

**Figure 2:**
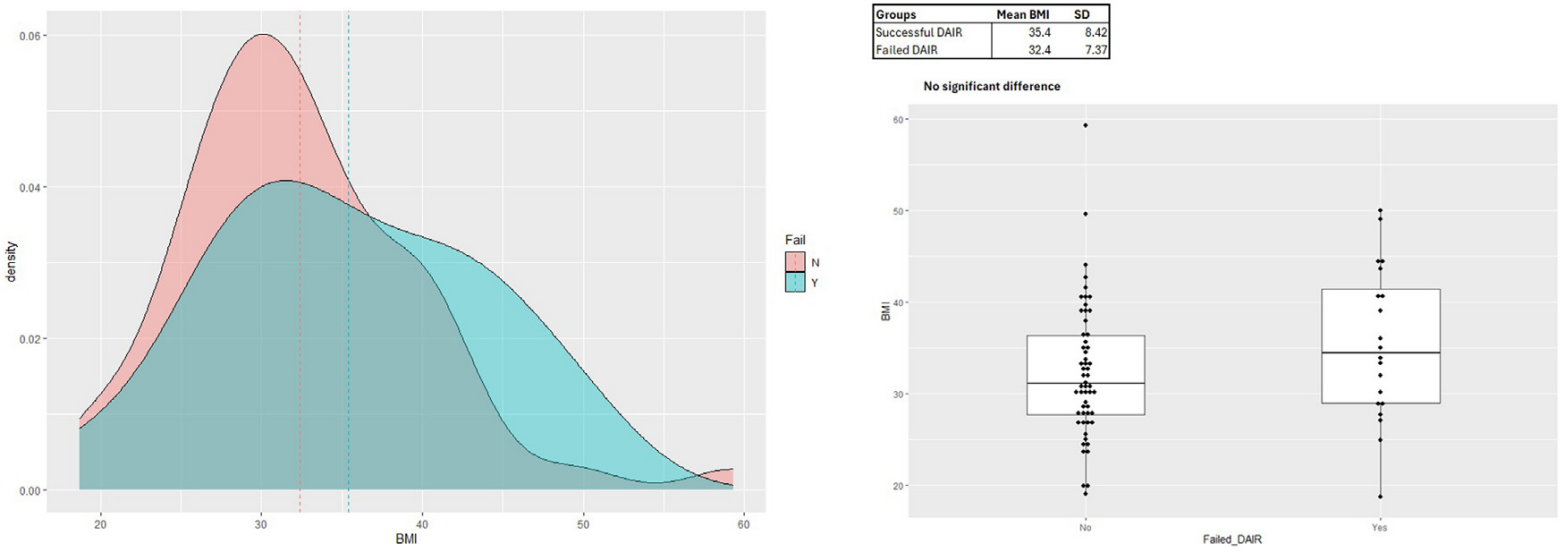
Graphical and box and whisker plot representations of BMI density distributions for failed and successful DAIR groups. There was no significant difference between groups.

**Figure 3:**
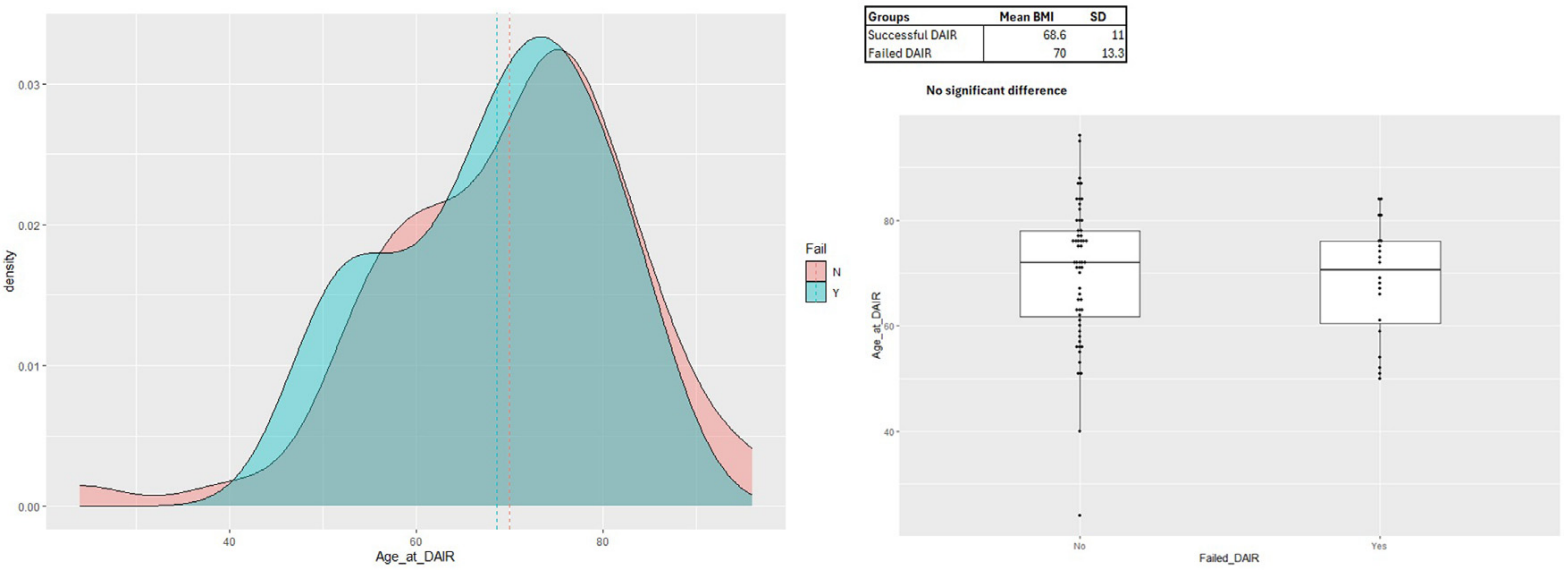
Graphical and box and whisker plot representations of age at the time of DAIR density distributions for failed and successful DAIR groups. There was no significant difference between groups.

DAIR failed in 50% of diabetic patients compared to 20% of nondiabetic patients (*P* = .015) (Fig. 1). Age at the time of DAIR, body mass index (BMI), and organism identification did not influence outcome (Figure 1, Figure 2, Figure 3) DAIR within 6 weeks of primary surgery was successful in 81% of patients, compared to 70% of DAIR successful if greater than 6 weeks from primary surgery (*P* = .312).

## Discussion

Bactisure was used in 26 of 76 hip or knee DAIR procedures at our institution during the study period. Overall, there was no statistically significant difference in infection eradication rate between patients who received Bactisure and those who did not. Similarly, within the knee cohort, there was no significant difference between groups. Within the hip cohort, there was a possible trend toward of improved outcomes when Bactisure was used, with 100% infection eradication; however, this was not statistically significant. We found no adverse effects of Bactisure use. A finding of note was the higher rate of DAIR failure in diabetic patients, which was statistically significant (*P* = .015). Furthermore, patients who underwent DAIR procedure within 6 weeks of their primary surgery had a greater success of infection eradication.

Published literature has previously investigated the efficacy of Bactisure and other irrigation solutions in vitro [[18], [19], [20],^22^]. Márquez-Gómez et al. [23] inoculated stainless steel discs with strains of methicillin-susceptible Staphylococcus aureus, methicillin-resistant S. aureus, Pseudomonas eruginosa, and Staphylococcus epidermidis. They then irrigated the inoculated discs with commonly used antiseptic solutions (10% and 3% povidone iodine, hydrogen peroxide, 3% acetic acid, and Bactisure) and sterile saline as a control. Assessing reduction in biofilm based on colony-forming unit counts, they found that all solutions tested showed a >90% decrease in colony-forming unit count but went on to recommend a sequential combination of antiseptic solutions. O’donnel et al. tested the efficacy of different antiseptic solutions against clinically relevant biofilms and found Bactisure and Povidone-iodine had a larger cumulative reduction in biofilm burden compared to the other solutions tested [19]. Christopher et al. investigated the effect of exposure time on the effectiveness of antiseptic solutions, of which Bactisure was one, and concluded that all tested solutions demonstrated eradication of all bacterial growth in less than 2 minutes except chlorhexidine [24]. Overall in vitro results appear promising; however, there is no clear evidence of increased effectiveness over more traditional antiseptics.

Very few in vivo studies evaluating the use of Bactisure have been reported. An industry-sponsored white paper by Hunter and Duncan [25] demonstrated a reduced bioburden and bacterial count within the surgical site following Bactisure lavage during DAIR or 1st of 2 stage revision for infection. Andriollo et al. reviewed the outcomes of DAIR/debridement, antibiotic pearls, and retention of the implant procedures with Bactisure use in 39 patients, demonstrating successful infection eradication in 87.3% of patients. However, no comparative cohort was included. This study represents the first study looking at in-vivo use of Bactisure as an adjunct to DAIR for hip and knee PJI, with comparison to a control group. The success rate of 74% in this study is commensurate with reports in the literature of success rates for DAIR ranging from 55.5% up to 90% with a recent systematic review by Longo et al. reporting an average of 71% [[26], [27], [28]].

Previous studies have reported additional risk factors contributing to failed DAIR such as age, BMI, diabetes, rheumatoid arthritis, malignancy, and immunosuppressive medications [29,30]. This study highlighted that for diabetic patients, the failure rate of DAIR procedures was significantly higher (diabetic patients failed DAIR in 50% of cases). This is similar to the findings of multiple studies reported in the literature [28,31,32]. Diabetic patients require greater consideration of appropriate management strategies, with consideration of more aggressive surgical intervention to minimize the requirement for further surgery. We are unaware of clinical guidelines to direct management for this high-risk patient group. A greater duration since time of primary surgery was associated with a higher failure rate of DAIR in this study, but this difference did not reach statistical significance. Analysis of BMI, American Society of Anesthesiologists, and age did not show any significant differences.

It is important to note that there were limitations with this study. There are inherent limitations due to its retrospective design. Given the many variables potentially contributing to success or failure of DAIR procedures, the outcomes presented would be more robust with a larger cohort of patients, particularly within the Bactisure group. The trend toward statistical significance in hip patients receiving Bactisure in encouraging; however, this group ultimately remains too small to offer a definitive answer. While a period of 12 months postoperatively is considered adequate for monitoring of recurrence, the long-term outcomes of these patients remain unknown. We will continue to follow this patient cohort and report longer-term results. The longer-term safety profile of Bactisure use is not clearly established; however, we are not aware of any reported adverse effects in the literature to date.

## Conclusions

While in vitro research suggests benefit of its use, clinical studies, to date, though promising have been scarce and have also not been conclusive. In this study, the addition of Bactisure to DAIR procedures did not demonstrate a statistically significant improvement in successful eradication of infection supporting our initial hypothesis. Bactisure use demonstrated no adverse effects within this cohort. Ultimately, larger prospective controlled studies in the future may be able to provide us with more definitive answers.

## CRediT authorship contribution statement

**Jonathan Quinn:** Writing – original draft, Project administration, Methodology, Investigation, Data curation, Conceptualization. **Bernard H. van Duren:** Writing – original draft, Visualization, Project administration, Methodology, Investigation, Formal analysis, Conceptualization. **Reshid Berber:** Writing – review & editing, Investigation. **Mark Higgins:** Writing – review & editing, Investigation. **Hosam E. Matar:** Writing – review & editing, Supervision, Investigation. **Andrew R. Manktelow:** Writing – review & editing, Supervision, Investigation. **Benjamin V. Bloch:** Writing – review & editing, Supervision, Methodology, Conceptualization.

## Conflicts of interest

B. Bloch is a paid consultant and speaker bureau for DePuy Synthes, Zimmer Biomet, and Ethicon; receives research support from DePuy Synthes (from 2017-2020); and is an editorial board member of Bone & Joint 360. A. R. Manktelow receives royalties and research support from Matortho; is a paid consultant and speaker bureau for Matortho, Zimmer Biomet, and Medacta; and is a council member of the British Orthopaedic Association. B. H. van Duren receives research support from Medacta International and is an editorial board member of Bone & Joint 360. R. Berber is an educational/speaker/general consultant for Johnson & Johnson MedTech and is a principle investigator for a study supported by Johnson & Johnson MedTech. All other authors declare no potential conflicts of interest.

For full disclosure statements refer to 10.1016/j.artd.2024.101593.

## Funding

B.H. van Duren is supported in part by the National Institute for Health and Care Research (NIHR) Leeds Biomedical Research Centre (BRC) (NIHR203331). The views expressed are those of the author(s) and not necessarily those of the NHS, the NIHR or the Department of Health and Social Care.

## Appendix A Supplementary data

Conflict of Interest Statement for Bloch

Conflict of Interest Statement for Matar

Conflict of Interest Statement for Quinn

Conflict of Interest Statement for Berber

Conflict of Interest Statement for Manktelow

Conflict of Interest Statement for van Duren

Conflict of Interest Statement for Higgins
