# Supplementary material for: Efficacy of Adjunctive Antiseptic Lavage Solution in Managing Acute Hip/Knee Prosthetic Joint Infection: A Comparative Study in a Tertiary Revision Center
Source: Arthroplast Today. 2025 Jan 21;31:101593. doi: 10.1016/j.artd.2024.101593 (PMC11794171; doi:10.1016/j.artd.2024.101593)
Supplement: Conflict of Interest Statement for Bloch [file mmc1.docx]

# INDIVIDUAL CONFLICT OF INTEREST STATEMENT

***American Association of Hip and Knee Surgeons***

(Adopted from the American Academy of Orthopaedic Surgeons disclosure statement)

The following form **must be filled out completely and submitted by each author (example, 6 authors, 6 forms).**

**All items require a response. If there is no relevant disclosure for a given item, enter "*None*.”**

**Manuscript Title: Efficacy of Adjunctive Antiseptic Lavage Solution in Managing Acute Hip/Knee Prosthetic Joint Infection: Comparative Study in a Tertiary Revision Centre**

1. Royalties from a company or supplier (The following conflicts were disclosed)

N/A

2. Speakers bureau/paid presentations for a company or supplier (The following conflicts were disclosed)

DePuy Synthes, Zimmer Biomet, Ethicon

3A. Paid employee for a company or supplier (The following conflicts were disclosed)

N/A

3B. Paid consultant for a company or supplier (The following conflicts were disclosed)

DePuy Synthes, Zimmer Biomet, Ethicon

3C. Unpaid consultants for a company or supplier (The following conflicts were disclosed)

N/A

4. Stock or stock options in a company or supplier (The following conflicts were disclosed)

N/A

5. Research support from a company or supplier as a Principal Investigator (The following conflicts were disclosed)

DePuy Synthes (from 2017-2020)

6. Other financial or material support from a company or supplier (The following conflicts were disclosed)

N/A

7. Royalties, financial or material support from publishers (The following conflicts were disclosed)

N/A

8. Medical/Orthopaedic publications editorial/governing board (The following conflicts were disclosed)

N/A

9. Board member/committee appointments for a society (The following conflicts were disclosed)

Editorial Board, Bone & Joint 360

**Each author must sign AND print or type his/her name, date and submit a separate form**

In addition, one BLINDED Conflict of Interest form (no author names used) should be submitted per manuscript with all author disclosures.


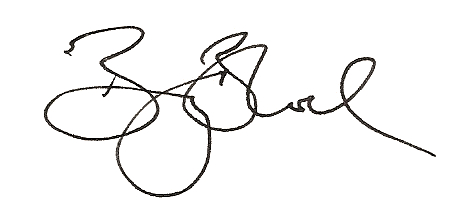


Benjamin Bloch 4^th^ Sept 2023

Author Name (Print or Type) Author Signature Date
