# Supplementary material for: Efficacy of Adjunctive Antiseptic Lavage Solution in Managing Acute Hip/Knee Prosthetic Joint Infection: A Comparative Study in a Tertiary Revision Center
Source: Arthroplast Today. 2025 Jan 21;31:101593. doi: 10.1016/j.artd.2024.101593 (PMC11794171; doi:10.1016/j.artd.2024.101593)
Supplement: Conflict of Interest Statement for Quinn [file mmc3.pdf]

INDIVIDUAL CONFLICT OF INTEREST STATEMENT

American Association of Hip and Knee Surgeons

(Adopted from the American Academy of Orthopaedic Surgeons disclosure statement)

The following form **must be filled out completely and submitted by each author (example, 6 authors, 6 forms). All items require a response. If there is no relevant disclosure for a given item, enter "*None.*"**

Manuscript Title: Efficacy of Adjunctive Antiseptic Lavage Solution in Managing Acute Hip/Knee Prosthetic Joint Infection: Comparative Study in a Tertiary Revision Centre

1. Royalties from a company or supplier (The following conflicts were disclosed) - NONE

2. Speakers bureau/paid presentations for a company or supplier (The following conflicts were disclosed) NONE

3A. Paid employee for a company or supplier (The following conflicts were disclosed) NONE

3B. Paid consultant for a company or supplier (The following conflicts were disclosed) NONE

3C. Unpaid consultants for a company or supplier (The following conflicts were disclosed) NONE

4. Stock or stock options in a company or supplier (The following conflicts were disclosed)NONE

5. Research support from a company or supplier as a Principal Investigator (The following conflicts were disclosed) NONE

6. Other financial or material support from a company or supplier (The following conflicts were disclosed) NONE

7. Royalties, financial or material support from publishers (The following conflicts were disclosed) NONE

8. Medical/Orthopaedic publications editorial/governing board (The following conflicts were disclosed) NONE

9. Board member/committee appointments for a society (The following conflicts were disclosed) NONE

Each author must sign AND print or type his/her name, date and submit a separate form

In addition, one BLINDED Conflict of Interest form (no author names used) should be submitted per manuscript with all author disclosures.

JONATHAN QUINN	[signature]	24/09/2024
Author Name (Print or Type)	Author Signature	Date
