# Supplementary material for: Efficacy of Adjunctive Antiseptic Lavage Solution in Managing Acute Hip/Knee Prosthetic Joint Infection: A Comparative Study in a Tertiary Revision Center
Source: Arthroplast Today. 2025 Jan 21;31:101593. doi: 10.1016/j.artd.2024.101593 (PMC11794171; doi:10.1016/j.artd.2024.101593)
Supplement: Conflict of Interest Statement for Berber [file mmc4.pdf]

INDIVIDUAL CONFLICT OF INTEREST STATEMENT

American Association of Hip and Knee Surgeons

(Adopted from the American Academy of Orthopaedic Surgeons disclosure statement)

The following form **must be filled out completely and submitted by each author (example, 6 authors, 6 forms). All items require a response. If there is no relevant disclosure for a given item, enter "*None.*"**

Manuscript Title: Efficacy of Adjunctive Antiseptic Lavage Solution in Managing Acute Hip/Knee Prosthetic Joint Infection: Comparative Study in a Tertiary Revision Centre

1. Royalties from a company or supplier (The following conflicts were disclosed) None

2. Speakers bureau/paid presentations for a company or supplier (The following conflicts were disclosed)
Educational/Speaker Consultancy for Johnson and Johnson Medtech.

3A. Paid employee for a company or supplier (The following conflicts were disclosed) None

3B. Paid consultant for a company or supplier (The following conflicts were disclosed)
General Consultancy for Johnson and Johnson Medtech

3C. Unpaid consultants for a company or supplier (The following conflicts were disclosed) None

4. Stock or stock options in a company or supplier (The following conflicts were disclosed) None

5. Research support from a company or supplier as a Principal Investigator (The following conflicts were disclosed)
Principle Investigator for a study supported by Johnson and Johnson Medtech

6. Other financial or material support from a company or supplier (The following conflicts were disclosed) None

7. Royalties, financial or material support from publishers (The following conflicts were disclosed) None

8. Medical/Orthopaedic publications editorial/governing board (The following conflicts were disclosed) None

9. Board member/committee appointments for a society (The following conflicts were disclosed) None

Each author must sign AND print or type his/her name, date and submit a separate form

In addition, one BLINDED Conflict of Interest form (no author names used) should be submitted per manuscript with all author disclosures.

Reshid Berber	R. Berber	24/09/24
Author Name (Print or Type)	Author Signature	Date
