# Supplementary material for: Efficacy of Adjunctive Antiseptic Lavage Solution in Managing Acute Hip/Knee Prosthetic Joint Infection: A Comparative Study in a Tertiary Revision Center
Source: Arthroplast Today. 2025 Jan 21;31:101593. doi: 10.1016/j.artd.2024.101593 (PMC11794171; doi:10.1016/j.artd.2024.101593)
Supplement: Conflict of Interest Statement for Manktelow [file mmc5.docx]

# INDIVIDUAL CONFLICT OF INTEREST STATEMENT

***American Association of Hip and Knee Surgeons***

(Adopted from the American Academy of Orthopaedic Surgeons disclosure statement)

The following form **must be filled out completely and submitted by each author (example, 6 authors, 6 forms).**

**All items require a response. If there is no relevant disclosure for a given item, enter "*None*.”**

**Manuscript Title: Efficacy of Adjunctive Antiseptic Lavage Solution in Managing Acute Hip/Knee Prosthetic Joint Infection: Comparative Study in a Tertiary Revision Centre**

1. Royalties from a company or supplier (The following conflicts were disclosed) Matortho

2. Speakers bureau/paid presentations for a company or supplier (The following conflicts were disclosed) Matortho

Zimmer Biomet and Medacta

3A. Paid employee for a company or supplier (The following conflicts were disclosed) Nil relevant

3B. Paid consultant for a company or supplier (The following conflicts were disclosed) Matortho. Zimmer biomet and Medacta

3C. Unpaid consultants for a company or supplier (The following conflicts were disclosed) Nil relevant

4. Stock or stock options in a company or supplier (The following conflicts were disclosed) Nil relevant

5. Research support from a company or supplier as a Principal Investigator (The following conflicts were disclosed) Matortho

6. Other financial or material support from a company or supplier (The following conflicts were disclosed) Nil relevant

7. Royalties, financial or material support from publishers (The following conflicts were disclosed) Nil relevant

8. Medical/Orthopaedic publications editorial/governing board (The following conflicts were disclosed) Nil relevant

9. Board member/committee appointments for a society (The following conflicts were disclosed)

Council member British Orthopaedic Association

**Each author must sign AND print or type his/her name, date and submit a separate form**

In addition, one BLINDED Conflict of Interest form (no author names used) should be submitted per manuscript with all author disclosures.

Andrew RJ Manktelow
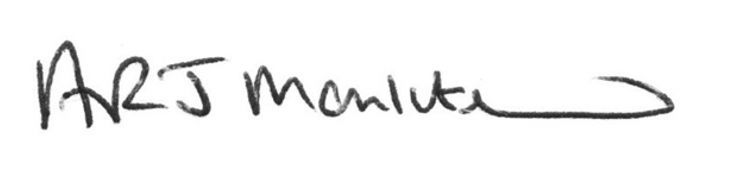


24.09.2024

Author Name (Print or Type) Author Signature Date
